# Biomarkers for predicting and monitoring the efficacy of cancer vaccines

**DOI:** 10.3389/fonc.2026.1904586

**Published:** 2026-07-24

**Authors:** Julia A. Remizova, Anastasia G. Volkova, Anna S. Makarova, Valentin V. Makarov, Vladimir S. Yudin, Anton A. Keskinov

**Affiliations:** Federal State Budgetary Institution «Centre for Strategic Planning and Management of Biomedical Health Risks» of the Federal Medical and Biological Agency, Moscow, Russia

**Keywords:** biomarkers, cancer vaccines, cell-based vaccines, DNA vaccines, immunotherapy, malignant neoplasm, mRNA vaccines, peptide vaccines

## Abstract

Cancer vaccines are a modern approach to personalized anti-tumor therapy for malignant neoplasms based on stimulating the patient’s immune system to target tumor cells. Many studies have demonstrated the impressive efficacy of anti-tumor vaccines in treating patients with different localization, type and stage of neoplasm. One of the major obstacles to the widespread implementation of cancer vaccines into clinical practice is the lack of reproducible biomarkers to evaluate and predict the efficacy of tumor vaccine therapy. The most commonly used methods for assessing a patient’s immune status are ELISA and flow cytometry, which make it possible to assess the activation of the patient’s immune system. However, multiple clinical trials have not yet demonstrated the reproducibility of these study results. This review includes description of efficacy biomarkers and methods for their evaluation used in a number of tumor vaccines’ clinical and preclinical trials, and discuss the challenges of applying the biomarkers in real-world clinical practice.

## Introduction: therapeutic anti-tumor vaccines and immunity

Immuno-oncology research has helped expand the possibilities to apply immunotherapeutic strategies in the treatment of malignant neoplasms (MN). Immunotherapy of malignant neoplasms is aimed at activating and enhancing the body’s natural anti-tumor immune response. This involves infiltration of the tumor and tumor microenvironment (TME) by cells of the adaptive (B- and T-cells) and innate (natural killer cells, eosinophils, basophils, mast cells, neutrophils, monocytes, macrophages and dendritic cells) immune systems, which leads to modulation of tumor progression (1–4). Tumor cells have several escape mechanisms from immune surveillance, such as defects in the antigen (Ag) presentation system and the recruitment of immunosuppressive cell populations (5–7). Taking into account the incomplete knowledge about the processes occurring at different stages of immunotherapy, this limits its possible widespread use in clinical practice.

As a promising approach in immunotherapy, therapeutic anti-tumor vaccines are capable of overcoming insufficient tumor immunogenicity (8), but their development is greatly complicated by selection and preparation of target Ags. Thus, anti-tumor vaccines can target tumor-associated Ags (TAA) or tumor-specific Ags (TSA) (9, 10). TAA is expressed in normal tissues and overexpressed in malignant tumors (9). Immunocompetent cells can recognize TAA as the body’s own Ags and remove them from the immune repertoire, as a result of which anti-tumor immune responses do not develop (11, 12). TSA is a neoantigen formed as a result of somatic mutations specific to each particular tumor (13). TSA rarely induce immune tolerance, which makes them better targets for tumor immunotherapy (13). However, the time of vaccine production, the costs of its development, and the subsequent creation of a pool of personalized neoantigens are major challenges for the large-scale implementation of the technology.

The history of the anti-tumor immunotherapy idea began with the observation that tumor regressed in some sarcoma patients who had survived an *Streptococcus pyogenes* infection (14, 15). A detailed study of the anti-tumor immunity activation led to the development of the therapeutic anti-tumor vaccination concept, which involves delivery of highly immunogenic Ag to antigen-presenting cells (APCs) and induction of stable CD4^+^ and CD8^+^ T-cell responses and long-lasting immunological memory (16–18).

TAA, which can be used as targets for vaccines, include non-mutated embryonic proteins expressed by tumors (NYESO-1, proteins of the MAGE-A family), human endogenous retroviruses, tissue differentiation Ags (MART-1, gp100) and overexpressed tumor Ags (HER2 in ovarian and breast cancer, carcinoembryonic Ag in epithelial tumors) (19). It is worth mentioning that neoantigens (neoAg), which are formed as a result of driver mutations of tumors and are typical for large groups of patients (BRAFV600E, KRASG12C, EGFRT790M and others) can also be used as targets (20). In other cases, Ags are specific for each individual patient and correspond to neoantigens, which arise as a result of rare mutations in tumor cells (somatic single nucleotide variants, insertions, deletions and gene fusions). Such vaccines, developed on the basis of the individual repertoire of the patient’s Ags, are classified as personalized therapy (19, 21).

Anti-tumor vaccines can be based on RNA and DNA, peptides and proteins, oncolytic viruses and cells - dendritic cells (DC), tumor cells, stem cells (SC). Despite the wide variety of vaccine platforms, many clinical trials have shown relatively low efficacy of anti-tumor vaccines in terms of clinical responses (8, 22, 23). However, existing achievements in this field have showcased the viability of tumor immunotherapy (24). In order to select patients who will respond to immunotherapy, a system of reliable biomarkers must be developed (25).

Studying the mechanisms of immunotherapy resistance is necessary to establish a comprehensive biomarkers system. Methods for assessing the patient’s immune status include phenotyping of cell populations using flow cytometry; enzyme-linked immunosorbent assay for assessing cytokine release (ELISPOT) and their profiling (ELISA); tetramer analysis of Ag-specific cellular responses; sequencing of T-cell (TCR) receptors to determine the immune repertoire, genetic organization and specificity (18, 26). Methods for assessing the immune status also include immunohistochemistry (IHC) and molecular genetic studies of tumor samples, if available (18).

This review is devoted to assessing predictive biomarkers for cancer immunotherapy on the basis of the patient’s immune status before prescribing vaccine therapy and in the course of the treatment. A literature search was performed in the Pubmed database. The search depth was 25 years.

Below is a brief description of the anti-tumor vaccine platforms which will be discussed in the review.

### Nucleic acid-based vaccines

Anti-tumor therapeutic vaccines based on messenger ribonucleic acid (mRNA) include three main types: non-replicating mRNA vaccines (nrRNA), self-amplifying mRNA vaccines (saRNA), and trans-amplifying mRNA vaccines (27, 28). nrRNA vaccines are synthetic analogues of mature mRNA, saRNA is the result of genetic engineering of viruses with the replacement of their genes with Ag sequences, and trans-amplifying mRNA has a replicase enzyme, which causes the replication of a two-component vector system which includes both saRNA and nrRNA (27, 29, 30). DNA vaccines are plasmids encoding tumor Ags (31). Ag encoded by a DNA vaccine can be presented using MHC class I and II, i.e. activation of both CD4^+^ and CD8^+^ T-cells is possible (32). Moreover, due to the double-stranded structure and the presence of CpG motifs, DNA vaccines themselves can be immunogenic and induce innate immune responses regardless of the encoded Ags translation (33).

### Peptide vaccines

Peptide anti-tumor vaccines include a 20–30 amino acid sequence representing TAA or TSA, or multiple epitopes against multiple targets (34). Several clinical trials of early peptide vaccines showed a lack of clinical benefit for patients (35), which was explained by the inability of the peptides and adjuvants used to provide adequate stimulation of Ag presentation. Whole proteins-based vaccines have also not been shown to be effective, which can be explained by less efficient processing and presentation of proteins by dendritic cells compared to short peptides (36).

### Neoantigen vaccines

Neoantigen vaccines target mutated tumor proteins and have great therapeutic potential due to the fact that host immune cells are able to recognize tumor neoantigens as foreign. This was demonstrated by the results of using immune checkpoint inhibitors (ICIs) in cancer with a high tumor mutational burden (TMB) (37, 38). Neoantigen vaccines include peptide-based, DNA-based, RNA-based, adenovirus-based and dendritic cell (DC)-based vaccines (39–42).

### Cell-based vaccines

Cell-based vaccines are mainly based on DCs and autologous or allogeneic tumor cells. The success of cell-based vaccines is demonstrated by the sipuleucel-T vaccine for the treatment of castration-resistant prostate cancer (NCT00065442), which was the first cancer vaccine approved by the US Food and Drug Administration (FDA) (43). This vaccine consists of autologous peripheral blood mononuclear cells loaded with prostatic acid phosphatase Ag fused to granulocyte-macrophage colony-stimulating factor (GM-CSF, an immune cell activator). The comparative study of effectiveness of sipuleucel-T and androgen-receptor signaling pathway inhibitors (ASPIs) in clinical practice indicates significantly longer survival after sipuleucel-T treatment (44).

DCs are antigen-presenting cells, which determines the main principle of action of DC anti-tumor vaccines: antigen capture, internalization and processing into peptides that are then presented in the context of MHC Class I and II molecules. As a result, activated T-cells become able to recognize (CD4^+^ T-cells), remember (memory T-cells) and eliminate (CD8^+^ T-cells) tumor cells (45).

Whole tumor cell (WTC) vaccines use whole or lysed tumor cells as a source of a broad spectrum of Ags (46). Autologous tumor cells can be obtained directly from the patient, which guarantees the presence of specific Ags and implies personalized vaccination. In contrast, allogeneic WTC vaccines are produced from tumor cells obtained from another person or using a tumor cell line. Such vaccines are a source of tumor-specific Ags and are suitable for patients from whom tumor sampling is not possible (46).

### Exosome-based vaccines

Exosomes are organelles secreted by a variety of cell types, including tumor cells and immune cells. Exosomes can contain proteins, lipids, conjugates, and nucleic acids synthesized within the cell (47, 48). Therefore, exosomes can promote intercellular signaling and regulation (49, 50). Exosomes are used as biomarkers for cancer screening and early diagnosis (51, 52). Exosomes derived from tumor tissues can be used not only as a platform for of functional RNAs delivery to target cells, but also, in combination with immunostimulatory agents, as an initiator of a brisk anti-tumor CD8^+^ T-cell response (53, 54). DC-derived exosomes (DEX) contain MHC I and II molecules and CD86, which are involved in the activation of CD4^+^ and CD8^+^ T-cells (53, 54). It is shown that DEX administration can induce T-cell activation and anti-tumor immune response *in vivo* (55).

### Oncolytic viruses

Vaccines based on oncolytic viruses (OV) are *in situ* vaccines, characterized by the induction and stimulation of an immune response directly at the tumor site using the entire available TAA repertoire. The therapeutic efficacy of such vaccines is due to the preferential replication in tumor cells followed by cytolysis (56). Efficacy of OV-based vaccines is demonstrated by talimogene laherparepvec (T-VEC), which is a modified herpes simplex virus 1 (HSV1), expressing GM-CSF, and is FDA-approved for the treatment of advanced malignant melanoma (57). However, the short half-life of oncolytic viruses and the limited viral replication and dissemination in TME limit the efficacy of vaccines of this type. For this reason, oncolytic viruses are usually used in combination with other therapeutic regimens (58).

The types of anti-tumor vaccines described above are the main representatives of this type of immunotherapy. However, vaccines that stimulate the immune response without delivering tumor Ags are also used. For example, vaccination with *Mycobacterium bovis* bacillus Calmette–Guérin (BCG) received approval for use in some types of bladder cancer (BC) (59).

It is therefore can be seen that clinical efficacy of anti-tumor vaccines currently has significant limitations, which motivate researchers to develop improved vaccine modifications, optimize their immunogenicity, develop more reliable delivery platforms and more effective adjuvants (24, 27, 34).

## Biomarkers for predicting the efficacy of cancer vaccines: Evaluation of the immune status of patients and tumor immunogenicity before vaccine therapy

The use of anti-tumor vaccines require careful preparation. This includes, firstly, the selection of target Ags and secondly, the stratification of patients for whom the vaccine will be effective. In both cases, examination of blood and tumor samples is required. Identification of patients suitable for the use of an anti-tumor vaccine includes accurate HLA typing, identifying Ag’s serological activity and assessment of the patient’s neoantigen repertoire (60–63). It is often necessary to analyze tumor or target antigens-associated gene expression. The status of the DNA mismatch repair (MMR) system or the presence of microsatellite instability (MSI) and tumor mutational burden (TMB) are also necessary information for making decisions on the use of anti-tumor immunotherapy, since they characterize potential immunogenicity of the tumor.

It is also worth mentioning the conventional division of tumors into “hot” and “cold”. The term “hot tumors” often denotes tumors with TME enriched in immune cells with anti-tumor potential, such as CD8+ T-cells. Tumors with a highly immunosuppressed TME, depleted of effector immune cells, are classified as “cold” (64).

**Table 1** lists methods for examining samples before using immunotherapeutic drugs, including anti-tumor vaccines of different types. In general, these methods are aimed at studying the activity of the patient’s immune system and characterizing the TME. The application of these methods to assess predictive biomarkers of anti-tumor vaccine efficacy is discussed below. However, it is shown by the literature review and the results of clinical studies that it is necessary to set sensitive threshold values ​​for these methods and develop new, more accurate and specific immune status evaluation techniques for a high-precision prediction of anti-tumor vaccination effectiveness.

**Table 1:** Studies conducted prior to the use of anti-tumor vaccines and their corresponding biomarkers.

Assessment method	Biomarker
IHC	“Immunoscore” (i0, i1, i2, i3, i4), based on the two lymphocyte populations assessment (CD3/CD45RO, CD3/CD8 or CD8/CD45RO) both in the tumor core and in the invasive margin
	TME infiltration with TILs (effector CD8^+^Т cells, CD8α/CD103 line DC, FoxP3^+^ Treg)
	PD-L1 expression in tumor-associated immune cells
WGS/WES/targeted sequencing	MSI/MMR status (MSI-H/dMMR, MSI-L/pMMR)
	TMB
	Tumor mutation status
	Tumor Ag-targets
RNA sequencing, RT-PCR	Gene expression
Flow cytometry	Blood T-cells phenotyping (CD3^+^, CD8^+^, cytotoxic Т lymphocytes, T-cell surface markers)

### TME infiltration

IHC analysis is used for detection of structural and functional proteins and their spatial localization within cells or extracellular compartments. This is achieved by immunolabeling with specific antibodies and results in cells phenotyping (65). As an example, consensus immunoscore was proposed as an indicator for colorectal cancer (CRC). This indicator of high prognostic value has 4 levels of gradation (66–68). Patient stratification is based on lymphocyte typing and assessment of the density and localization of CD3 and CD8 lymphocytes populations in the TME. Data on the type, density, and location of immune cells within the tumor samples turned out to be better predictor of patient survival than the UICC-TNM staging system (69, 70). Tumor infiltrating lymphocytes (TILs) have been assessed in autologous tumor cell vaccine study in melanoma patients. IHC was used to assess the infiltration of metastatic lesions by CD4^+^, CD8^+^ and CD20^+^ cells before and after vaccination. It was shown that diffuse infiltrates were associated with more extensive tumor necrosis (71). The complexity of TME of vulvar high-grade squamous intraepithelial lesions patients, receiving peptide vaccine, was assessed using spatial transcriptomics. Key immune populations enriched in patients with better clinical response included CD4+CD161+ effector T cells and chemotactic CD4+ and CD8+ T cells (72).

### MSI/dMMR status

MSI is manifested when there is a deficiency of the DNA mismatch repair (dMMR) system, which is accompanied by a pronounced mutational profile. MSI is used as a predictive marker in ICI immunotherapy of cancer (73). MSI/MMR status is assessed using fragment analysis, IHC or Next-generation sequencing (NGS). It is most often studied for gastrointestinal tumors (74–76). NGS allows to determine MSI status simultaneously with a complete profile of changes in the tumor genome or exome (77–79). The predictive value of the MSI/dMMR marker for the efficacy of immunotherapy is due to the fact that MSI-high (MSI-H) tumors harbor multiple non-editable mutations that create target neoantigens for T-cells (80, 81). For example, MSI status was determined in patients with glioblastoma (GBM) before multipeptide vaccine therapy, but the results of study did not reveal significant differences in survival associated with MSI. This indicates that it is still difficult to judge the applicability of MSI/dMMR as a biomarker of the efficacy of anti-tumor vaccine therapy (82). However, it was shown that most of gastroesophageal adenocarcinoma patients, who responded to immunotherapy with PD-1 blockade, have MSI-H tumors (83).

### PD-1/PD-L1 expression

Programmed cell death 1 (PD-1) protein is expressed by tumor-infiltrating immune cells. Its ligand (PD-L1) is expressed by tumor cells and APCs. Interaction of these immune checkpoint proteins negatively regulates the anti-tumor immune response (84). It appears from this that inhibition of this checkpoint promotes activation of anti-tumor immunity and elimination of malignant cells. Predictive significance of PD-1/PD-L1 expression has been confirmed in numerous studies of antiPD-L1/anti-PD-1 drugs (85, 86). However, in the studies of the anti-PD-1 inhibitor nivolumab for non-small cell lung cancer (NSCLC) patients (87) and a multipeptide vaccine in melanoma patients (88) this indicator could not statistically significantly predict the clinical response. PD-L1 expression is determined mainly by the IHC method in clinical practice (89, 90).

### Tumor mutational burden

Tumor mutational burden (TMB) is defined as the ratio of somatic mutations per megabase (mut/Mb) of the studied genomic sequence and is considered, depending on the value of the ratio, as an indicator of possible presence of immunogenic neoantigens (91). There is a correlation between the TMB level and the clinical response to drugs based on Ab blockade of CTLA4 (92) or PD-L1/PD-1 pathway (93). TMB acts as a predictive (bio)marker for ICI immunotherapy of various oncological diseases (94–96). This indicator varies among cancer types and there is no set prognostic threshold for TMB across all tumor types. However, in some patients with previously treated, unresectable or metastatic solid tumors, TMB ≥10 mutations/Mb is associated with better clinical responses to therapeutic anti-PD-1 antibodies (97). TMB is assessed by WGS, WES or targeted panel sequencing. However, using WES to determine TMB is conventionally considered the “gold standard” (98–100). To promote reproducibility and comparability of TMB estimation, a software tool was proposed (101).

The results of using TMB as a predictive biomarker in oncovaccines studies are discordant: in a trial of a peptide vaccine for GBM 7 of 173 patients showed TMB levels of ≥10 mut/Mb, but their elevated TMB was not associated with vaccine efficacy (82). In a study of the efficacy of a multipeptide vaccine in combination with an anti-PD-L1 agent, a significant positive correlation between 9-month progression-free survival (PFS) and TMB before vaccination was observed in the melanoma cohort and a positive trend was defined in the NSCLC cohorts (102). An interesting observation is described in a case report (NCT03468244) of a patient with advanced esophageal squamous cell carcinoma, who received personalized mRNA vaccine. The patient’s TMB was measured before and after vaccination and it was shown that blood TMB (bTMB) decreased from 7.63 to 1.53 mut/Mb after 4 cycles of treatment, which indicates suppression of tumor cell growth (103).

### HLA typing

Human leukocyte antigen (HLA), also known as the major histocompatibility complex (MHC), is a set of genes encoding cell surface molecules, which present own and foreign peptides/antigens (104–108). MHC class I molecules present peptide fragments of endogenously degraded own or foreign proteins, including TAA and TSA, to the T-cell receptors (TCRs) of CD8^+^ cytotoxic T lymphocytes (CTLs) (106, 109–111). Tumor cells demonstrate specific expression of the class II MHC complex (tsMHS-II), the level of which may be associated with the efficacy of anti-PD-1/anti-PD-L1 therapy (112–117). The correlation between tsMHC-II expression and clinical efficacy of ICI was found using such methods of analysis as IHC and RNA sequencing (113, 118). HLA alleles determine the neoantigen repertoire of the tumor, which is presented to T-cells. The presence of HLA-LOH (loss-of-function), which impairs Ag presentation, contributes to immune evasion. Therefore, HLA typing is an important step in predicting the immunogenicity of neoantigens (119).

HLA typing was performed in clinical trials of neoantigen peptide vaccines - EGFRvIII-specific Rindopepimut (CDX-110), H3K27M-vac and NEO-PV-01. No HLA subtype was associated with increased PFS or OS (102, 120, 121). Nevertheless, in a large phase III clinical trial of the RINDOPEPIMUT (CDX-110) vaccine correlation analysis revealed an association of HLA types B18, A25 and D11 with OS or PFS in individual analyses. However, the sample size was small in this trial and the association was not observed consistently in the populations under analysis (122).

### Tumor mutation status

As already mentioned, the main sources of neoantigens are somatic genomic alterations such as single nucleotide variants (SNVs), insertions, deletions, as well as structural variants: fusions, inversions, translocations, duplications/amplifications (123). The listed sources of neoantigens are identified using WGS, WES and targeted sequencing data analysis, which is important for the development of effective anti-tumor vaccines and patient stratification for immunotherapy (124–127). It was found using WES in a study of a multipeptide vaccine for the treatment of metastatic solid tumors that the presence of at least one copy of the germline variant of the *APOE4* gene in the tumor is positively correlated with 9-month PFS in patients with melanoma and negatively correlated with PFS in patients with NSCLC (102). However, a relatively small number of neoantigens have been identified to date that have the ability to induce specific T-cell responses. This makes prediction of neoantigen immunogenicity a critical point for increasing the effectiveness of vaccines of this type.

Tumor mutational status can be used as a biomarker even for tumors with a relatively low mutational burden. In a study of vaccine targeting triple-negative breast cancer (TNBC) researchers used WES to identify between 4 and 20 cancer neoantigens for each patient and produced a neoantigen DNA vaccine, which caused neoantigen-specific immune responses in the majority of patients (128). The creation of the RCC neoantigen-targeting vaccine involved WES of tumor tissue and peripheral blood to identify neoantigens and RNA-seq of tumor to confirm the expression of putative neoantigens (41).

### Gene expression analysis

RNA-sequencing data analysis is most often used for differential gene expression detection in tumors, tumor biomarkers discovering and monitoring (129–131). For a similar purpose, the reverse transcription-polymerase chain reaction (RT-PCR) gene expression analysis is used (132). More widespread applications of RNA-sequencing include the detection of gene fusions (RNA-CaptureSeq) (133), diverse non-coding RNAs (134), mutations (135). A modern method for single-cell transcriptome analysis (Single-sell-RNA-seq, scRNA-seq) allows to obtain information on gene copy number variations (CNV) (136). Gene expression is most often used as a biomarker to determine the immune status of a tumor (126, 137, 138).

For example, intratumor expression of inflammatory marker genes prior to vaccination was analyzed using the NanoString nCounter platform in a study of a multipeptide vaccine for the treatment of patients with metastatic solid tumors. A trend for positive correlation was found between the expression of certain genes and PFS in melanoma and BC cohorts (102). R132H *IDH1* mutant variant was found to be associated with reduced effector CD8^+^ T-cells gene expression in GBM patients (139). PCR was used to assess the expression of *EGFRvIII* target mutations in tumor cells in a clinical trial of the peptide vaccine Rindopepimut (CDX-110). It was shown that the absence of *EGFRvIII* expression in tumor cells at relapse is associated with a longer OS and PFS (120). However, this finding was not supported by a subsequent phase III clinical trial (122). Medium and high expression of *BNIP3L* was linked with longer overall survival of melanoma patients in the trial of the cell vaccine AGI-101H (140). Specific cell types, genes and pathways were shown to be associated with vaccine response in a study of the MUC1 tumor antigen vaccine (141).

However, in some cases expression biomarkers did not have a relationship with the outcome of immunotherapy. *MAGEA3* expression profiling by PCR before peptide MAGE-A3-specific vaccine treatment of melanoma patients did not reveal significant association between biomarker expression and clinical response to vaccine therapy (88). Similarly, no relationship was observed between clinical responses and pre-treatment expression profile of *CD8A, CD274, KDR* and T cell-inflamed gene expression profile of 15 biomarkers in patients with advanced HCC, receiving DNA plasmid vaccine (142).

### Blood T-cells phenotyping

Flow cytometry is a powerful immunoassay (IA) which helps to analyze large numbers of single cells with multiple parallel probes to obtain phenotypic and functional characterization of cell subsets (65). Moreover, flow cytometry allows the assessment of T-cell specificity using *in vitro* Ag stimulation followed by cytokine profiling.

The association between presence of tumor-specific polyfunctional T-cells in the blood and the response to anti-tumor vaccines therapy, including multipeptide vaccines with DNA adjuvant in melanoma, have been assessed in several studies. However no significant correlation was found (143). It was shown in a trial of a multipeptide vaccine for metastatic solid tumors therapy that the percentage of naive CD8+ T-cells before vaccination was higher in melanoma patients with progression. NSCLC patients who achieved PFS of 9 month had a lower percentage of plasmacytoid DCs than patients who progressed (102).

### Cell-free DNA fragments characterization (NGS)

The study of cell-free DNA (cfDNA) circulating in the blood is a minimally invasive and quite sensitive assay that is now gradually becoming a tool for quantitative monitoring of tumor genomic alterations (144), including MSI status (145–148). For example, it was shown using liquid biopsy approaches that circulating tumor DNA (ctDNA) level decreases after one cycle of ICI therapy (149–151). At the same time, cfDNA is a promising biomarker for anti-tumor vaccines efficacy assessing. In particular, there is published research on cfDNA integrity assessment before and after the use of a personalized peptide vaccine. A decrease in cfDNA integrity in NSCLC has been described, as well as association between high levels of cfDNA integrity before/after vaccination and a higher OS (152). Similar results were obtained in a retrospective study assessing the integrity of cfDNA after the use of a peptide vaccine in endometrial cancer (153).

## Biomarkers identified during and after vaccine therapy

The clinical efficacy of anti-tumor therapy, including vaccine therapy, is determined by its ability to prolong patient survival and slow down disease progression. At the same time, anti-tumor vaccines are known to induce immune reactions that lead to pseudoprogression of tumors (121, 154). The efficacy of immunotherapeutic strategies is thought to be primarily determined by TME infiltration by immune cells which provide anti-tumor immunity (155). In many cases, TME is predominantly immunosuppressed and is characterized by depletion of effector T- and NK-cells and accumulation of regulatory T-cells, type 2 T helper cells, CD4^+^ T-cells, tumor-associated macrophages (TAM) and myeloid-derived suppressor cells (MDSC) that suppress the development of immune responses (156–158). The following review will discuss biomarkers identified through monitoring immune responses to anti-tumor vaccine therapy and the methods used to detect these biomarkers. **Table 2** summarizes the most commonly used methods for investigating biomarkers of immune status in patients receiving anti-tumor vaccine therapy.

**Table 2:** Studies conducted to monitor the efficacy of tumor vaccines and relevant biomarkers.

Estimation method	Biomarker
IA (ELISPOT, ELISA, etc.)	IFNy and other cytokines in peripheral blood and in TME
Flow cytometry	Phenotyping of T-cells in peripheral blood and TME (CD3^+^, CD8^+^, CTL, T-cells surface markers)
Flow cytometry, tetramer assay	MHC-peptide complexes in peripheral blood and tumor samples
NGS, PCR	Clonality and diversity of T-cell receptors in peripheral blood and TME
RNA-seq, PCR	Gene expression in the tumor

### Cytokines and Ag-specific responses of Ab and T-cells

The ELISpot immunoassay technique is a universal tool for quantifying Ag-specific T-cells by analyzing the production of cytokines, most commonly IFNγ. In the traditional ELISpot assay, T-cells are cultured with putative peptide epitopes for 10 days, followed by immunoassay detection of IFNγ. ELISpot quantitative output correlates with the potency of the Ag-specific T-cell response, although it cannot be used to determine the absolute number of Ag-specific T-cells. In particular, ELISpot has been used to assess cellular immune response after anti-tumor vaccines immunotherapy (159–161). IA is the primary method for assessing immune reactions. However, the reproducibility of IA is a problem that precludes the use of immunoassay reactions as unambiguous biomarkers of anti-tumor vaccine efficacy.

There is a subset of studies which link ELISpot to meaningful clinical outcomes. In a phase II clinical trial of the Rindopepimut vaccine (CDX-110) in combination with temozolomide (TMZ), blood IA detected *EGFRvIII*-specific Ab in 6 of 18 GBM patients with *EGFRvIII* mutation, which correlated with OS growth (162). *EGFRvIII*-specific Ab level was significantly higher in cases where the *EGFRvIII* genetic variant was no longer expressed at relapse (120) and in those receiving higher doses of concomitant TMZ therapy (163). However, the correlation between *EGFRvIII*-specific Ab levels and clinical outcomes found in early trials of the Rindopipemut + TMZ combination was not confirmed in a large-scale phase III trial (ACT IV) that was terminated after interim analysis due to lack of clinical efficacy of the combination (122). The phase II ReACT clinical trial of Rindopepimut (CDX-110) in combination with bevacizumab showed that a peak *EGFRvIII*-specific Ab titer ≥1:12800 is associated with longer OS (164). Correlation of Ag-specific Ab responses detected by ELISpot with patient survival has been demonstrated in two clinical trials of vaccines based on autologous tumor cells loaded with bi-shRNA furin/GM-CSF used to treat advanced solid tumors (165, 166). Immune response detected by ELISpot in the patients after different cell vaccines therapy was shown to be correlated with OS in PCa (167), in GBM (168) and melanoma (140), as well as with OS (166) and PSF (169) in ovarian cancer (OC). Ag-specific T-cells were detected by IA in the peripheral blood of patients with solid tumors treated with a DC vaccine loaded with 3 to 13 TAAs overexpressed in the respective patient’s tumor (170). For several cancer types, a correlation was found between clinical outcome and the proportion of TAAs whose mRNA was used to immunize autologous DCs and which induced Ag-specific T-cell responses (170).

On the other hand, phase III clinical trials of peptide vaccines against gp100 and MAGE-3 for advanced melanoma therapy failed to detect or confirm correlation between the development of anti-peptide Ab immune reactivity and clinical responses, as was demonstrated in (171, 172). A lack of significant correlations was also showed in an early study of a peptide vaccine for NSCLC therapy of increased IgG- and CTL-responses with clinical efficacy (173). However, in a later study Ag-specific and T-cell immune responses were shown to be correlated with higher PFS and OS (174). In contrast, trials of the same vaccine in NSCLC, biliary tract cancer (BTC) and pancreatic cancer (PCa) using IA have found significant correlations of C-reactive protein, serum amyloid A, IL-6, albumin and T-cell responses with OS (175–177). This indicates that immune responses monitoring by IA should not be limited to determining Ab or T-cells. For example, apolipoprotein A-I (APOA1) and chemokine (C-C motif) ligand 17 (CCL17) were shown to be predictive for both immune response and overall survival in a study of a multipeptide vaccine for renal cell carcinoma treatment (178). ELISpot has been used in numerous studies of peptide anti-tumor vaccines as a method to assess T-cell responses, but the result of this assay often did not correlate with clinical outcomes (102, 154, 179, 180).

There are similar challenges in using IA to detect biomarkers of immune response for other types of cancer vaccines. Clinical efficacy of a cell-based vaccine based on allogeneic tumor cells in PCa was evaluated by IA of serum GM-CSF. Peak levels are shown to be associated with systemic eosinophilia but not with clinical responses (181), or showed no significant associations with any indices (182). It was also the case for DC-based vaccines for melanoma treatment (71, 183–185), or for DC vaccines against CRC (186). No association between tumor-specific T-cell responses detected by ELISpot and clinical outcomes was found in a phase II and IIb clinical trial (TRAVERSE) of a modified cowpox virus vaccine Pexa-Vec (JX-594), expressing GM-CSF and β-galactosidase, in hepatocellular carcinoma (HCC) (187, 188).

Cytokine response can be assessed also in multi-antigen vaccines studies. When studying a tri-antigen vaccine targeting IGFBP-2, HER2, and IGF-IR in participants with non-metastatic breast cancer, researchers compared maximal IFNγ response magnitude of each antigen individually and found no dominance of one antigen over the others (189). The total vaccine response in this study was considered as the sum IFN-γ magnitude from all antigens (189).

The main limit of ELISpot as an oncovaccines efficacy biomarker is that it tracks immunogenicity rather than clinical benefit. ELISPOT protocols evaluate the activity of T cells and do not characterize ability of T-cells to penetrate the tumor microenvironment and overcome local immunosuppression. Moreover, the method is critically dependent on the quality of isolation, freezing and thawing of the patient’s blood cells.

### Phenotyping of T-cells in peripheral blood and TME

As previously said, flow cytometry is originally an immunological research method and is therefore used to evaluate cellular immune responses to immunotherapy of different types. However, similarly to IA, the reproducibility of this method is an issue, which creates difficulties in identifying reliable biomarkers of immune response to anti-tumor vaccine therapy.

Here are some examples of the use of cytometry in studies of cancer vaccine efficacy. *EGFRvIII*-specific responses to peptide vaccination in GBM showed no correlation with clinical outcome (163). Similarly, lack of correlations has been shown when peptide vaccines are used to treat breast cancer (190) and ovarian cancer (191) or when melanoma patients are treated with mRNA-vaccine (192), DC-based vaccines (184) or Langerhans cells (193), as well as when treating CRC with a DC vaccine (186). In a phase III clinical trial of a peptide vaccine targeting the gp100 protein for the treatment of melanoma, blood level of CD4^+^FOXP3^+^ T-cells was determined using flow cytometry and was shown to be significantly higher in patients with clinical responses to therapy than in non-responders, but this statistical difference was not influenced by the vaccine (171). In the efficacy study of the neoantigenic peptide vaccine IDH1-vac for the therapy of patients with GBM (NOA16) it was demonstrated that cellular immune responses detected by flow cytometry were associated with tumor pseudoprogression (154).

Conversely, a number of studies have successfully identified statistically significant correlations of cytometric results with clinical outcomes. T-cell response, detected by flow cytometry, was significantly correlated with median survival in a study of a personalized multipeptide anti-GBM vaccine (82). Postvaccine phenotyping of peripheral blood immune cells from patients with metastatic solid tumors, treated with a multipeptide personalized vaccine, showed that the levels of memory CD8^+^ T-cells and stem cell signatures were positively correlated with 9-month PFS in melanoma and 6-month PFS in BC. Conversely, the percentage of naive CD8^+^ T-cells was positively correlated with melanoma progression, and lower levels of plasmacytoid DCs in patients with NSCLC were negatively correlated with 9-month survival (102). In the same study, IHC was used to assess major pathological responses (MPRs), which refers to residual tumor viability <5%, evaluated by the proportion of tumor cells in the biopsy sample. Thus, the presence of MPRs in patients with melanoma was associated with higher PFS (102). A statistically significant positive correlation between the PFS duration and the cumulative frequencies of antitumor antigen-specific CD8+ T cells was observed in patients with metastatic NSCLC receiving allogenic DC-based vaccine PDC^∗^lung01 (194).

A series of clinical trials of a peptide vaccine targeting human papillomavirus (HPV) E6 and E7 Ags were conducted in patients with grade 3 vulvar intraepithelial neoplasia (VIN3) caused by HPV type 16 (HPV16) (179, 180, 195). Using flow cytometry, a study (179) evaluated cytokine production by tumor-infiltrating T-cells after vaccination and it was shown that higher levels of the immune response cytokines IFNγ, IL-5, TNFα and IL-10 are associated with smaller tumor size as well as clinical response to vaccine therapy. Finally, this work demonstrates a high probability that patients with a strong response to the first dose of vaccine will develop a complete clinical response (179). HPV16-specific T-cell responses were also assessed using cytometry, and it was shown that higher blood levels of HPV16-specific IFNγ producing CD4^+^ and CD8^+^ T-cells with high proliferative activity are associated with complete clinical response (195). A later study demonstrated a relationship between immune response and tumor size: CD4^+^CD25^+^Foxp3^+^ T-cell populations prevail in large tumors with a smaller amplitude of response to therapy, whereas CD4^+^CD25^+^Foxp3^-^ T-cells predominate in small tumors that responded to treatment (179). Similarly, the CD4^+^CD25^+^Foxp3^-^ T-cell response was stronger in patients with complete tumor regression and with a relatively short case history (179).

Evaluation of the efficacy of multipeptide vaccination of RCC patients by flow cytometry in phase I and II trials showed a strong association between low percentage of T-regulatory cells in the peripheral blood of patients before vaccination and multiple T-cell responses to the vaccine. At the same time, the number of suppressive myeloid cells from the CD14^+^HLA-DR^-/low^ and CD11b^+^CD14^-^CD15^+^ populations was negatively correlated with OS (178). In the same study, the expression of TCR-ζ by T-cells was assessed using flow cytometry and a trend toward a positive association between TCR-ζ expression and survival was shown (178). In a study of a peptide MAGE-A3-specific vaccine for melanoma therapy, higher expression of the proteins 41BB, CTLA-4 and LAG3 by MAGE-A3-specific CD8^+^ T-cells was found to be related to a lack of clinical response to therapy. Conversely, a decrease in the expression of these proteins was significantly associated with the presence of a response in patients (88).

When comparing samples obtained before and after administration of a cell-based autologous vaccine in patients with melanoma, IHC analysis of metastatic lesions revealed association between infiltration of samples with CD4^+^, CD8^+^, and CD20^+^ cells and extensive tumor necrosis (71). IHC was used to evaluate the infiltration of the vaccine administration site during treatment of patients with PCa and RCC with an autologous tumor cell-based vaccine, and it was reported that detection of signs of delayed-type hypersensitivity (DTH) was associated with an improvement in PFS (182, 196).

In situations where Ag-specific assays cannot be used, an alternative is to investigate T-cell or Ab responses using DTH test. To determine the specificity of the immune response, the DTH assay should include appropriate comparison elements, including common Ag, for example, influenza, Candida, tetanus anatoxin. In a study of a cell-based vaccine for patients with PCa, infiltrates of mononuclear cells and eosinophils were found in the area of vaccine administration. This was associated with DTH, characteristic manifestations of which were observed in patients who did not show clinical signs of disease after vaccination (182). DTH reactions are actively investigated in clinical trials of cancer vaccines (71, 162, 183–185, 196, 197). As a biomarker, DTH has demonstrated correlation with clinical response in RCC (196), metastatic RCC (198) and GBM (162). Interestingly, in the trial of a neoantigenic peptide vaccine, targeting *EGFRvIII*, GBM patients developed DTH at higher but not standard TMZ dose, which was also accompanied by higher OS and PFS in this clinical subgroup (163). In a study of a peptide vaccine in NSCLC, the presence of a skin reaction at the site of vaccine administration was associated with an increase in the PFS (174).

### Evaluation of MHC: peptide complexes in peripheral blood and TME

A modified approach of flow cytometry for CTL detection, the tetramer assay, uses major histocompatibility complex (MHC) multimers linked to antigenic peptides to detect and quantify T-cells, which are specific for a particular peptide-MHC complex and play a central role in a response to an Ag-specific cancer vaccine (65). TCR binding to the peptide-MHC complex is a major characteristic of an Ag-specific T-cell, therefore peptide-MHC multimers conjugated to fluorochromes enable the visualization of Ag-specific T-cells by flow cytometry and the phenotypic characterization of these populations (65). For example, it was found in a clinical trial of an allogeneic cell-based vaccine for therapy of PCa that the MesoA2 (531–539) tetramer titer after treatment is correlated with OS (181). Studies of a DC vaccine loaded with gp100 and tyrosinase for the treatment of metastatic uveal melanoma revealed tetramer-positive T-cells in peripheral blood. In this population, CD4^+^ cells were detected before vaccination and CD8^+^ cells - only after vaccination. Number of T-cells increased significantly under the action of DC, but no significant correlation of this parameter with clinical outcome was found (185). In other clinical trials, the tetramer assay did not detect any changes in T-cell reactivity against vaccine Ags on top of immune responses detected by other methods (193). A tetramer assay performed to evaluate the efficacy of a DC vaccine targeting pp65 CMV for the treatment of GBM showed dynamic changes in tetramer binding intensity: increasing after the first three vaccinations and decreasing after the next vaccination. However, no statistically significant correlation of this parameter with clinical response was found (199).

### Clonality and diversity of TCRs in peripheral blood and TME

Each T-cell-mediated response depends on TCR recognition of peptide-MHC complexes. NGS methods are used to identify a repertoire of TCRs and BCRs unique to each individual T- or B-cell clone, which allows characterizing the course of the disease and making some predictions (200–202). The complementarity determining region 3 (CDR3) sequence is a TCR region responsible for contact with the peptide-MHC complex and Ag recognition, which may inform about the clonal hierarchy of T-cells in norm and in pathology (201). TCR sequencing allows monitoring the progress of the disease, detecting responses to therapy, identifying minimal residual disease and searching for potential immunotherapeutic biomarkers (65). At the same time, TCR sequence data alone do not provide information about the Ag-specific character of the T-cell response. Therefore, a study combining Ag stimulation of T-cell culture and subsequent TCR sequencing has become a promising approach to overcome the limitations of other methods for assessing the anti-tumor immune response.

Peripheral and intratumoral changes in TCR frequency or clonality were found in GBM patients who received DC-based vaccine therapy. A significant correlation between the number of TILs determined by TCR sequencing and PSF and OS was also detected (203). Moreover, sequencing of TILs TCR isolated from peripheral blood and tumor samples gave information about the pool of overlapping TCR sequence between blood and tumor. A higher degree of such overlap was statistically significantly associated with greater clinical responses (203). A study of TCR clonality before and after administration of a Langerhans DC-based vaccine for the treatment of patients with melanoma showed that patients with relapse of disease tended to have less pronounced changes in TCR clonality than patients without relapse, but no significant correlations were found (193). The correlation between epitope spread detected by TCR sequencing and PFS was shown when treating metastatic solid tumors patients with a personalized multipeptide vaccine (102). In (42) T cell receptor sequencing was used to trace the origin and longevity of RNA neoantigen vaccine-induced T cell clones. Combination of single-cell T cell receptor sequencing data with scRNA-seq data helped to compare the expansion, differentiation state, and phenotype between various clonotypes in melanoma (204), but such multi-modal approach has yet to be applied to the study of the effectiveness of cancer vaccines.

Clonality metrics vary by sample source, timing, sequencing depth, and analysis method, so they are not yet standardized enough to serve as a standalone surrogate endpoint.

### Gene expression in tumor cells

The expression of inflammatory biomarker genes was assessed using the NanoString nCounter platform targeting expression panel in a Phase Ib clinical trial of a multipeptide vaccine for the treatment of patients with metastatic solid tumors. It was found that the expression of genes participating in inflammatory reactions in tumor cells after vaccination was positively correlated with PFS in melanoma and tended to be positively correlated with the TMB level (102). Using microarray gene expression profiling, an expression signature of 84 genes was detected in patients receiving a peptide MAGE-A3-specific vaccine for melanoma or NSCLC, and higher expression of these genes was observed in patients who responded to vaccine therapy. This signature predicted complete or partial response to MAGE-A3 vaccine therapy with maximum sensitivity and specificity values, 0.79 and 0.69, respectively (205).

**Supplementary Table 1** presents a list of clinical trials that showed statistically significant associations between the indicators described in the sections above and clinical outcomes.

The described spectrum of correlations between levels of biological analytes and clinical outcomes of cancer patients who have received anti-tumor vaccines does not currently allow identification of candidate biomarkers of immune response for further validation and application in clinical practice. There are methods to detect the immune response to anti-tumor vaccines, but quantitative assessments of such immune response do not always predict the efficacy of vaccine therapy, and repeated studies in some cases may not confirm previously identified relationships between immune and clinical responses (122, 172). Advanced research to find and validate biomarkers of vaccine efficacy is highly relevant, but is currently limited due to various challenges: development and scaling up of the multistep process of obtaining different types of anti-tumor vaccines, organization and conduct of clinical trials. At the same time, the problems of identifying markers of the immune status of patients need to be solved as soon as possible due to intensive efficacy and safety research of new immunotherapeutic options.

### Biomarkers of cancer vaccines efficacy in preclinical trials

Although preclinical studies are limited by the fact that model systems cannot fully reproduce human diseases, such studies are justified in terms of obtaining and processing biological samples. This sometimes allows for the discovery of promising biomarkers for the effectiveness of the therapy under study in multi-method research. Particularly when studying tumor vaccines in model animals, researchers always have access to tumor samples after therapy. For example, Cordner R. and colleagues found in a study in GL26 model mice that the *IDH1R132H* mutation, which frequently occurs in GBM, reduces the efficacy of DC vaccine therapy but induces an endogenous immune response to the corresponding neoantigen (139). Moreover, it was shown that the survival of mice overexpressing wild-type *IDH1* was more than doubled after DC vaccination compared to control animals. In contrast, GBMs with a localized response to DC therapy in the form of increased CD8^+^ T-cell levels could demonstrate loss of *IDH1* expression, which predicted significantly shorter survival (139).

The study of a vaccine based on induced pluripotent stem cells (iPSCs) combined with valproic acid (VPA) in BALB/c and C57BL/6 mice with TNBC demonstrated improved survival and reduced tumor volume, which correlated with the generation of CD4^+^, CD8^+^ T-сells and CD44^+^CD62L^-^CCR7^low^CD127^low^ memory effector T-cells and with a reduction in the populations of CD4^+^CD25^+^FoxP3^+^ T-regulatory cells, Arg1^+^CD11b^+^Gr1^+^ and Arg1^+^CD11b^+^Ly6^+^ myeloid-derived suppressor cells (MDSC) in the tumor, as determined by flow cytometry (206). Moreover, the transformation of immunosuppressive TME into immunoreactive TME has been shown by reducing the levels of CD4^+^CD25^+^FoxP3^+^ T regulatory cells and MDSC, Arg1^+^CD11b^+^Gr1^+^pre-MDSC and Arg1^+^CD11b^+^Ly6^+^gramMDSC after vaccination (206).

Interestingly, *in situ* vaccine in gel form is able to induce repolarization of tumor-associated macrophages (TAM) toward the M1 phenotype, which in turn is associated with increased vaccine efficacy and improved survival in preclinical and clinical studies (207).

## Discussion

Biomarkers of the effectiveness of oncovaccines are necessary for identification of likely responders, monitoring of immune activity and improvement of trial design. These problems can potentially be solved by studying the activity of the patient’s immune system and characterizing the TME before prescribing immunotherapy, as well as in the course and after treatment. Predictive biomarkers, that have already found application in late-phase clinical trials, include TME infiltration, MSI, PD-1 expression and TMB. The most widely used monitoring biomarker is production of cytokines by T-cells (ELISpot). Nevertheless, none of these methods is a reliable standalone surrogate for survival or tumor control. The described methods for assessing biomarkers are most often used in combination with each other. This is true for assessments both before and during (or after) vaccination therapy. The integration of artificial intelligence (AI) into cancer vaccine development is revolutionizing this field, including predicting patient responses (208). For oncovaccines, AI-based biomarkers are promising mainly for patient selection, neoantigen target discovery, and response prediction, but the evidence is still largely indirect from broader immuno-oncology rather than vaccine-specific prospective trials (209).

Statistically significant correlation between biomarker quantities and the PFS/OS or other clinical outcomes was shown in many cases. These results suggest a relationship between the induction of an immune response and the clinical activity of a vaccine. However, there is a body of examples of lack of associations with clinical outcomes for each type of biomarker. This may be due to such limitations of reviewed studies as small number of patients and a limited follow-up time for OS assessment. Another problem it the variability of protocols, which leads to difference in the results across labs and operators. At present, the standardization of candidate biomarkers usage is poor. Even for such widely used measures as TMB, TME infiltration, PD-1 expression and T-cell response (ELISpot) prognostic thresholds are no set.

The studies described above demonstrate high heterogeneity of the tumor immunophenotype, and at the same time make it clear that our knowledge of the dynamic events occurring in tumors and TME during the immune response mediated by anti-tumor vaccines is limited. Most studies include only two time points that are usually many weeks or months apart. At the same time, critical events that distinguish therapy-responsive tumors from unresponsive tumors occur within days of therapy initiating (210). Consequently, in the context of assessing the immunophenotype of MN, it is important to obtain tumor samples both at earlier time points and more frequently during treatment. Ideally, serial tumor biopsies are suitable for these research tasks. This would allow to detail the development of anti-tumor immune response reactions, or to trace the formation of resistance. But in actual practice, such a large number of tissue biopsy specimens are rarely available. It is envisioned that liquid biopsy could be used as an additional, minimally invasive method to assess response to immunotherapy in real time. As an example, in (42, 211) peripheral blood T cell receptor sequencing was performed from pre-vaccination to approximately 4 years post-vaccination. Serum protein levels of 96 markers were assayed in patients with advanced melanoma after administration of a peptide vaccine targeting IDO and PD-L1 in combination with nivolumab (212). In this study increase in serum levels of CCL3, CCL4, and TNFα were shown to be biomarkers of long progression-free survival. Research (213) shows correlations between immune changes in the tumor immune microenvironment and peripheral blood in patients with PC, receiving a vector-based vaccine.

The described studies demonstrate that existing methods of assessing a patient’s immunophenotype are not always useful in predicting the efficacy of immunotherapy treatment. The landscape of immune responses is largely individualized, this creates obstacles to discover patterns in the modulation of these responses due to immunotherapeutic interventions. Nevertheless, anti-tumor vaccines are increasingly used in clinical practice and, despite the challenges, there are prospects for developing new vaccine platforms and identifying new ways to stratify patients for participation in clinical trials. Cancer vaccines represent one of the most promising and active areas of medical innovation, so more and more vaccine therapies are expected to become available and, as a consequence, biomarkers of their efficacy are expected to be identified.
