# Supplementary material for: Biomarkers for predicting and monitoring the efficacy of cancer vaccines
Source: Front Oncol. 2026 Jul 24;16:1904586. doi: 10.3389/fonc.2026.1904586 (PMC13448456; doi:10.3389/fonc.2026.1904586)
Supplement: Supplementary file 1 [file DataSheet1.docx]

Supplementary materials:

Table S1. Clinical trials of antitumor vaccines and identified biomarkers – immune response indicators, statistically significantly correlated with the clinical efficacy of vaccine therapy.

| **Cancer vaccine** | **Malignant neoplasm (MN)** | **Biomarker(s)** | **The observed biomarker correlations** | **Estimation method** | **Source** |
| --- | --- | --- | --- | --- | --- |
| Cell-based vaccine − Autologous/semi-autologous (mixture with allogeneic cell line) irradiated tumor cells | Metastatic renal carcinoma | DTH | The presence of DTH signs is correlated with survival | Examining of induration at the vaccine administration site | (200) |
| Cell-Based Vaccine − Allogeneic PCa cell lines CG8020 and CG2505, secreting GM-CSF | PCa | HLA-A2/Meso(20-28) and HLA-A2/Meso(531-539) | Level of MesoA2 (531-539) after treatment, detected by tetramer titration, is correlated with OS | Flow cytometry: avidity test using HLA-A2 tetramer assay | (183) |
| Cell-based vaccine − GVAX − GM-CSF expressing irradiated tumor cell lines Panc 10.05 and Panc 6.05, or GVAX+CRS-27 secreting mesothelin in infected APCs | PCa | Quantities of Ag-specific CD8^+^ T-cells | The quantities of Ag-specific cells are positively correlated with longer OS | IA | (168) |
| Neoantigen peptide vaccine − Rindopepimut (CDX-110) − *EGFRvIII* | GBM | Titers of serum Ag-specific Abs | Correlation with patient OS | IA | (163) |
|  |  | Ag-specific T-cells | Patients with an Ag-mediated DTH had longer OS and PFS than patients without DTH | IA |  |
| Neoantigen peptide vaccine − Rindopepimut (CDX-110) − *EGFRvIII* | GBM | Titers of serum Ag-specific Abs | Peak Abs titer values ≥1:12 800 is associated with longer OS | IA | (165) |
| Peptide vaccine - peptides were selected based on the patient's existing immunity before vaccination (up to 4 peptides) by IgG titers specific for each of 31 different candidate vaccines (12 peptides for HLA-A2, 14 peptides for HLA-A24, 9 peptides for HLA-A3 supertype and 4 peptides for HLA-A26) | NSCLC | Integrity of cell-free DNA | High cfDNA integrity (above the median value) before/after vaccination is positively correlated with OS | Real-time PCR | (153) |
| Peptide vaccine - peptides were selected based on the patient's existing immunity before vaccination (up to 4 peptides) by IgG titers specific for each of 31 different candidate vaccines (12 peptides for HLA-A2, 14 peptides for HLA-A24, 9 peptides for HLA-A3 supertype and 4 peptides for HLA-A26) | Endometrial cancer | Integrity of cell-free DNA | The biomarker was evaluated before and after vaccination. The integrity of cfDNA after vaccination is significantly correlated with OS | Real-time PCR | (154) |
| Cell-based vaccine - Vigil® Ovarian (gemogenovatucel-T, previously called FANG®) - autologous cells with rhGMCSF cDNA and bifunctional shRNA targeting furin | Solid tumors | Ag-specific T-cells concentrations | The presence of T-cell responses correlated with longer survival | ELISPOT | (169) |
| Cell-based vaccine - Vigil® Ovarian (gemogenovatucel-T) (FANG®) - autologous cells with rhGMCSF cDNA and bifunctional shRNA targeting furin | OC | Ag-specific T-cells concentrations | Biomarker level was evaluated before and after vaccination. 98% of the initial blood samples showed no immune activity against the autologous tumor cell population before Vigil. All samples tested showed induction of immune reactivity after Vigil vaccination, which correlated with higher PFS | ELISPOT | (166) |
| Multipeptide vaccine - peptides were selected based on the patient's existing immunity prior to vaccination (up to 4 peptides) by IgG titers specific for each of 31 different candidate vaccines (12 peptides for HLA-A2, 14 peptides for HLA-A24, 9 peptides for HLA-A3 supertype and 4 peptides for HLA-A26) | NSCLC | Ag-specific Ab concentrations | A correlation with longer PFS and OS has been shown | IA | (174) |
|  |  | Skin test response | The presence of a skin reaction is associated with higher PFS | Visual assessment of the injection site |  |
| Multipeptide vaccine - peptides were selected based on the patient's existing immunity prior to vaccination (up to 4 peptides) by IgG titers specific for each of 31 different candidate vaccines (12 peptides for HLA-A2, 14 peptides for HLA-A24, 9 peptides for HLA-A3 supertype and 4 peptides for HLA-A26) | NSCLC | T-cell responses | Evaluated before and after vaccination and are shown to be significant predictors of survival | ELISPOT | (175) |
|  |  | C-reactive protein (CRP) and serum amyloid A (SAA) concentrations | Evaluated before and after vaccination and are shown to increase significantly after vaccination. Significant predictors of survival: higher plasma CRP level before vaccination is a significant predictor of low OS | ELISA |  |
|  |  | IL-2, IL-4, IL-5, IL-6, IFN-γ concentrations | IL-6 level is a significant predictor of survival | ELISA |  |
| Multipeptide vaccine - peptides were selected based on the patient's existing immunity prior to vaccination (up to 4 peptides) by IgG titers specific for each of 31 different candidate vaccines (12 peptides for HLA-A2, 14 peptides for HLA-A24, 9 peptides for HLA-A3 supertype and 4 peptides for HLA-A26) | BTC | Ag-specific Ab concentration | Evaluated before and after vaccination. Number of peptides used is positively correlated with OS | IA | (176) |
|  |  | CRP and SAA concentrations | Evaluated before and after vaccination and are shown to be positive prognostic factors for OS | ELISA |  |
|  |  | IL-2, IL-4, IL-5, IL-6, IFN-γ, albumin concentrations | Evaluated before and after vaccination. IL-6 and albumin levels are shown to be prognostic factors: low IL-6 level and high albumin level are associated with longer OS | ELISA |  |
| Multipeptide vaccine - peptides were selected based on the patient's existing immunity prior to vaccination (up to 4 peptides) by IgG titers specific for each of 31 different candidate vaccines (12 peptides for HLA-A2, 14 peptides for HLA-A24, 9 peptides for HLA-A3 supertype and 4 peptides for HLA-A26) | PCa | Ag-specific Ab concentration | Evaluated before and after vaccination. High level of specific IgG is associated with a favorable prognosis | IA | (177) |
|  |  | CRP and SAA concentrations | Evaluated before and after vaccination. Pre-vaccination SAA level is negatively correlated with OS. Patients with higher levels of CRP and SAA before vaccination had a poor prognosis | ELISA |  |
| Multipeptide vaccine; 19 is the median number of peptides included in a single vaccine | GBM | MGMT methylation | Associated with longer OS. Evaluated before vaccination | WGBS | (82) |
|  |  | CD4^+^ and CD8^+^ T-cell response | The presence of a T-cell response in peripheral blood is associated with a significantly higher median OS | Flow cytometry |  |
|  |  | The Karnofsky performance status scale | Correlated with OS. Evaluated before vaccination | Medical history taking |  |
| Neoantigen peptide vaccine - NEO-PV-01 contains 20 personalized neoantigen peptides | Metastatic solid tumors (melanoma, NSCLC, BC) | TMB | TMB value is positively correlated with 9-month PFS in melanoma | WES | (102) |
|  |  | Epitope quality - a unique score assigned to each candidate epitope based on its binding affinity, allele-specific expression, and proteasomal cleavage potential (falls on a 0-1 scale, with higher scores corresponding to the increased likelihood of presentation) | Vaccine peptide epitope quality scores (sum of all HLA class I epitopes in vaccine peptides) are correlated with TMB | HLA-PCR |  |
|  |  | Expression of inflammatory marker genes in tumors | Positively correlated with PFS in melanoma (evaluated after vaccination) | NanoString nCounter |  |
|  |  | Infiltration of T-, B-, NK- and myeloid cells | A proportion of peripheral memory CD8^+^ T-cells and levels of stem memory T-cell signatures in the tumor are positively correlated with 9-month PFS in melanoma patients and with 6-month PFS in patients with BC (assessed after vaccination). The proportion of naive CD8^+^ T-cells before and after vaccination is positively correlated with melanoma progression. Lower level of plasmacytoid DCs in patients with NSCLC is negatively correlated with 9-month PFS. | Flow cytometry |  |
|  |  | *APOE4* mutations | The presence of at least one copy of an inherited variant of the *APOE4* gene is associated with higher 9-month PFS in melanoma and lower 9-month PFS in NSCLC | WES |  |
|  |  | Epitope spreading | Correlated with PSF | Single cell TCR sequencing |  |
|  |  | Major Pathological Responses (MPRs <5%) | In melanoma, the presence of MPR is associated with longer PFS | IHC cellularity assessment of tumor biopsy specimens |  |
| The multipeptide vaccine - a synthetic HPV16-SLP E6/E7 long-peptide vaccine (consists of a pool of 9 overlapping E6 peptides and 4 overlapping E7 peptides) | VIN | HPV16-specific CD4^+^ and CD8^+^ T-cells | CD4^+^ T-cells blood level is correlated with clinical response | Flow cytometry | (195) |
|  |  | T-cell proliferative responses | Enhanced proliferative response is associated with clinical response | The lymphocyte stimulation test |  |
| The multipeptide vaccine - a synthetic HPV16-SLP E6/E7 long-peptide vaccine (consisting of a pool of 9 overlapping E6 peptides and 4 overlapping E7 peptides) | Grade 3 vulvar intraepithelial neoplasia caused by HPV16, VIN3 | Production of cytokines (IFNγ, IL-5, TNFα and IL-10) | Negatively correlated with VIN3 lesion size and depth of response to vaccine therapy | Flow cytometry | (179) |
|  |  | Ag-specific CD4^+^ CD25^+^ Foxp3^+^ and Foxp3^-^ T-cells | The presence of Ag-specific T-cells is correlated with tumor lesion size: larger lesions (VIN) had relatively higher numbers of CD4^+^CD25^+^Foxp3^+^ T-cells, and relatively small lesions had infiltration of CD4^+^CD25^+^Foxp3^-^ T-cells. Similarly, the relative CD4^+^CD25^+^Foxp3^-^ T-cell response was stronger in patients with complete clinical response (complete tumor regression) and with relatively short disease duration | Flow cytometry |  |
| DC-based vaccine, loaded with 3-13 specific TAAs | Solid tumors (GBM and NSCLC) | Rate of Ags that induce Ag-specific T-cell response in peripheral blood | There is some positive correlation between the rate of Ags that induce Ag-specific T-cell response and clinical efficacy | IA | (170) |
| Cell-based vaccine - cytomegalovirus (CMV) pp65 RNA-pulsed autologous DCs | GBM | Ag-specific Ab levels | Abs levels are positively correlated with OS | ELISPOT | (169) |
|  |  | DC migration to lymph nodes draining the vaccination site (VDLN) | The intensity of DC migration is positively correlated with PFS and OS | Gamma camera (GE Infinia Hawkeye) |  |
| Multipeptide vaccine - IMA901 consists of several TAAs: 9 HLA-A*02 and 1 HLA-DR | RCC | Ag-specific T-cells in peripheral blood | Retrospectively found in subjects who had a response to vaccine with multiple Ags; for these patients disease control (disease stabilization or partial response) was recorded, compared to subjects who were found to have specific Abs to only one Ag or for whom no response to therapy was recorded | ELISPOT | (178) |
|  |  | Percentage of T regulatory cells | A low percentage of T regulatory cells before vaccination is correlated with multiple T-cell responses to the vaccine | Cell phenotyping using flow cytometry |  |
|  |  | Age | Significantly associated with fewer multiple Ags vaccine responses | Medical history taking |  |
|  |  | Prior treatment with cyclophosphamide (Cy) | The presence of prior Cy therapy is associated with longer OS | Medical history taking |  |
|  |  | Rate of Ags that induce Ag-specific T-cell response in peripheral blood | The rate of Ags that induce a response is positively correlated with OS | ELISPOT |  |
|  |  | MDSC4 and MDSC5 cell counts | The number of MDSC4 (CD14^+^HLA-DR^-/lo^) and MDSC5 (CD11b^+^CD14^-^CD15^+^) is negatively correlated with OS | Cell phenotyping using flow cytometry |  |
|  |  | Serum concentrations of APOA1 and CCL17 | Serum concentrations of APOA1 and CCL17 before vaccination are positively correlated with immune responses and multipeptide responses | Multiplex luminex technologies (Human MAP v1.6/Millipore) |  |
| Peptide vaccine - HER2/ neu-specific vaccine | BC | Epitope spreading | Epitope spreading, as detected by Ag-specific T-cell assay, is negatively correlated with serum TGF-ß levels (elevated in BC patients) | ELISPOT | (190) |
| Peptide vaccine - p53 SLP vaccine consists of 9 synthetic overlapping peptides containing amino acids 70-235 of the wt-p53 protein | OC | Swelling of the vaccine injection site | Magnitude of the swelling of the injection site is significantly positively correlated with a number of p53-specific T-cells. | Visual assessment | (191) |
| Peptide vaccine - GV1001 with 16-mer catalytic subunit of human telomerase reverse transcriptase (hTERT) | Adenocarcinoma of the pancreatic ducts | Initial clinical stage | Significantly associated with OS | Medical history taking | (doi: 10.1016/S1470-2045(14)70236-0) |
|  |  | CA19-9 blood level | Positively correlated with OS | Electrochemiluminescent immunoassay |  |
|  |  | ECOG somatic status | Inversely correlated with OS | Medical history taking |  |
| Peptide vaccine - MAGE-A3 (GlaxoSmithKline Biologicals S.A., Belgium) includes recombinant MAGE-A3 protein administered with proprietary immunostimulant AS15 (GSK) | Melanoma | Expression of 41BB, CTLA4 and LAG3 proteins by MAGE-A3-specific CD8^+^ T-cells | Evaluated before and after vaccination. Expression of 41BB, CTLA4 and LAG3 proteins was significantly increased in patients who did not respond to vaccine therapy and decreased in responding patients | Flow cytometry | (88) |
| Peptide vaccine - MAGE-A3 (GlaxoSmithKline Biologicals S.A., Belgium) includes recombinant MAGE-A3 protein administered with proprietary immunostimulant AS15 (GSK) | Melanoma, NSCLC | Gene expression | Expression signature of 84 genes, predominantly characterized by higher levels of gene expression in responding patients | Microarrays, RT-PCR | (205) |
| Cell-based vaccine - autologous DCs loaded with autologous whole tumor cell lysate | GBM | Anamnestic characteristics | Strong trend toward worse OS for patients over 35 years old. Complete resection is associated with higher PFS | Medical history taking | (doi: 10.1158/1078-0432.CCR-07-4875) |
| Cell-based vaccine - monocyte-derived DCs loaded with 4 HLA class I and 6 tumor peptides | Melanoma | Eosinophil count | Evaluated before and after vaccination. The occurrence of eosinophilia after vaccination is significantly associated with long-term survival of patients. Vaccine injection site reactions is associated with eosinophilia. | Clinical blood analysis | (doi: 10.1172/jci.insight.91438) |
| Cell-based vaccine - monocyte-derived or naturally circulating autologous DCs loaded with TSA gp100 and tyrosinase | Melanoma | Flu-like symptoms | Significantly correlated with the presence of tetramer-positive CD8^+^ T-cells or a TAA-specific functional T-cell response, as well as with a longer OS | Visual examination | (doi: 10.1097/  CJI.0000000000000127) |
|  |  | Injection site reactions | A correlation was found between injection site reactions and the presence of tetramer-positive CD8^+^ T-cells or a TAA-specific functional T-cell response, as well as with longer OS | Visual examination |  |
| Cell-based vaccine - AGI-101H - allogeneic whole-cell gene-modified vaccine (secretes the cytokine Hyper-IL6 (H6) and its soluble receptor sIL-6R, gp80) from human melanoma cell lines Mich-1 and Mich-2 | Melanoma | *BNIP3L* expression | Positively correlates with the OS index, with the expression of genes related to autophagy | RNA-seq | (140) |
|  |  | Presence of anti-BNIP3L/NIX Abs | Positive correlation with favorable clinical outcome | IA |  |

Abbreviations: PPC - pancreatic cancer, GBM - glioblastoma, NSCLC - non-small cell lung cancer, OC - ovarian cancer, EC - endometrial cancer, BCC - bile duct cancer, RCC - renal cell carcinoma, BC - bladder cancer, VIN3 - grade 3 vulvar intraepithelial neoplasia.
